# The Effects of Intranasal, Inhaled and Systemic Glucocorticoids on Intraocular Pressure: A Literature Review

**DOI:** 10.3390/jcm11072007

**Published:** 2022-04-03

**Authors:** Dries Wijnants, Ingeborg Stalmans, Evelien Vandewalle

**Affiliations:** 1 Department of Ophthalmology, University Hospitals UZ Leuven, Herestraat 49, 3000 Leuven, Belgium; ingeborg.stalmans@uzleuven.be (I.S.); evelien.vandewalle@uzleuven.be (E.V.); 2 Biomedical Sciences Group, Department of Neurosciences, Research Group Ophthalmology, KU Leuven, Herestraat 49, 3000 Leuven, Belgium

**Keywords:** glucocorticoids, safety profile, intranasal administration, inhaled administration, systemic administration, intraocular pressure, steroid response

## Abstract

Topical glucocorticoids are a well-known risk factor of intraocular pressure (IOP) elevation in one third of the general population and in up to 90% of glaucomatous patients. Whether this steroid response is caused by intranasal, inhaled or systemic glucocorticoids, is less known. This study presents an overview of the current literature on the topic, thereby providing guidance on when ophthalmological follow-up is indicated. A literature study was performed in Medline, and 31 studies were included for analysis. Twelve out of fourteen studies discussing intranasal glucocorticoids show no significant association with an elevated IOP. Regarding inhaled glucocorticoids, only three out of twelve studies show a significant association. The observed increase was either small or was only observed in patients treated with high inhaled doses or in patients with a family history of glaucoma. An elevated IOP caused by systemic glucocorticoids is reported by four out of the five included studies, with one study reporting a clear dose–response relationship. This review concludes that a steroid response can be triggered in patients treated with systemic glucocorticoids. Inhaled glucocorticoids may cause a significant IOP elevation when administered in high doses or in patients with a family history of glaucoma. At present, there is no evidence for a clinically significant steroid response caused by intranasally administered glucocorticoids.

## 1. Introduction

Glaucoma is defined as a chronic progressive optic neuropathy with corresponding visual field defects and structural changes at the optic nerve head [1]. The most important risk factor for glaucoma development and progression is an elevated intraocular pressure (IOP), and depending on the cause of IOP elevation, different disease entities are described. Most glaucoma cases present as primary open angle glaucoma, in which the eye shows an elevated IOP with an open anterior chamber angle, without any underlying condition. Nevertheless, a smaller portion of patients present with secondary glaucoma, where an underlying cause for the IOP elevation can be identified. Treating the cause in such patients can prevent further glaucomatous damage to the optic nerve. Multiple causes of secondary IOP elevation have been identified, most importantly ocular inflammation and trauma, pigment dispersion and exfoliation, neovascularization, dense cataract formation, corneal pathologies and the use of glucocorticoids [2].

Since 1951, a steroid response is known as the ability of glucocorticoids to increase IOP [3]. However, the mechanisms by which this phenomenon is established still remain unclear to date. Three contributing factors have been identified. First, glucocorticoids have been demonstrated to alter the trabecular meshwork microstructure by causing cross-links in the actin fiber network [4]. Second, they stimulate the deposition of extracellular matrix components such as collagen and fibronectin in the juxtacanalicular region, contributing to an increased outflow resistance [5]. Finally, steroids reduce the breakdown of substances in the trabecular meshwork by inhibiting cellular phagocytotic activity, reducing arachidonic acid metabolism and reducing the activity of degradation enzymes such as metalloproteinases, stromelysin and tissue plasminogen activator [6]. All of these mechanisms cause an increase in aqueous humor outflow resistance in the trabecular meshwork, which is the key factor in the pathophysiology of glucocorticoid-induced IOP elevation.

Whether or not an individual patient is susceptible to develop a steroid response depends on both drug-related and patient-related factors. The administered dose and duration of glucocorticoid intake play an important role, and due to different pharmacokinetic and pharmacodynamic properties, different glucocorticoid classes have different risks of developing a steroid response [7]. Dexamethasone is a potent glucocorticoid and therefore a more frequent cause of a steroid response [8]. Prednisolone is considered safer, although associations with a higher IOP were also described [8]. Glucocorticoids with the lowest effect on IOP are Fluorometholone, Medrysone, Rimexolone and Loteprednol [8].

Only one third of the general population is a steroid responder, showing an increased IOP after using topical glucocorticoids for two weeks or more [9,10,11], which reflects the interindividual differences in susceptibility. In contrast to the general population, the percentage of steroid responders rises to more than 90% for patients with pre-existing primary open-angle glaucoma [10,11]. In the pediatric population, the incidence of a steroid response is comparable to the general adult population, with some studies even describing a more frequent occurrence of the phenomenon in children [8,12]. Steroid response in children has an earlier onset and a more rapid progression than in adults, with some individuals developing an increased IOP after only one day of glucocorticoid intake [8]. Moreover, glaucomatous damage to the optic nerve can be more severe than in the adult population [13]. Considering that a steroid response can develop rapidly without obvious symptoms, it is crucial that clinicians have a proper knowledge of the possible harming effects of glucocorticoids in order to detect an elevated IOP or glaucomatous damage to the optic nerve in an early stage.

In contrast to this well-established steroid response caused by topical ocular glucocorticoids, it is much less clear whether glucocorticoids administered by other routes also cause a steroid response. Since intranasal glucocorticoids are the main treatment of various inflammatory otolaryngeal and nasopharyngeal conditions, such as different phenotypes of rhinitis, sinusitis, and associated headaches, the question arises of whether the ocular side-effects are also caused by glucocorticoids administered by this route [14,15]. The aim of this study is to present a clear overview of the existing literature on the effects of intranasal, inhaled and systemic glucocorticoids on IOP up until 2022 and to provide guidance on when additional monitoring of IOP is indicated.

## 2. Methods

**Figure 1:**
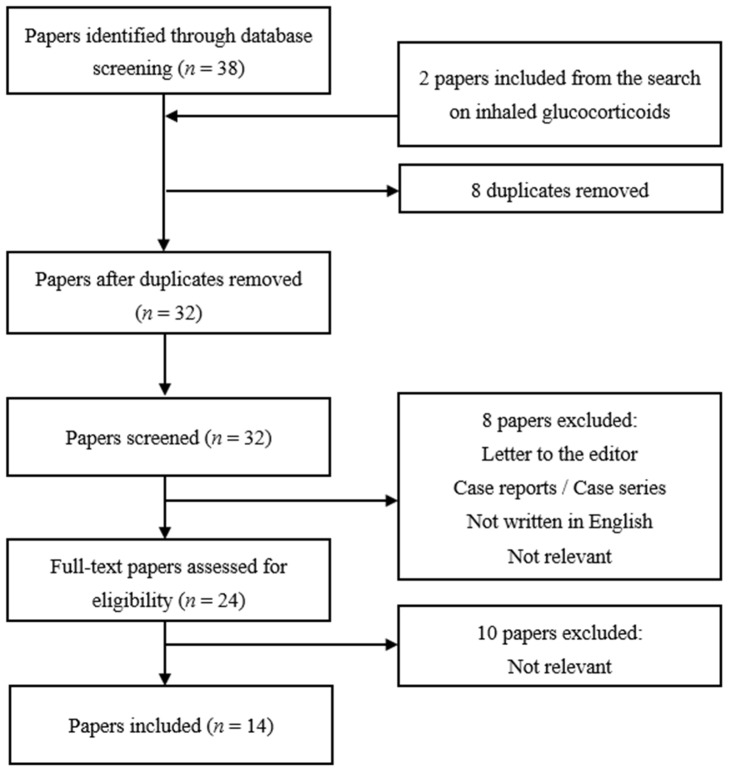
Study selection chart for intranasal glucocorticoids.

**Figure 2:**
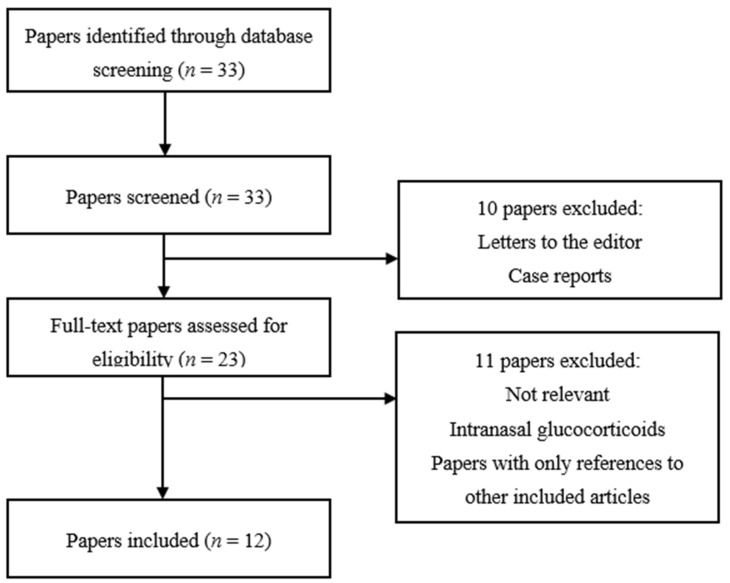
Study selection chart for inhaled glucocorticoids.

**Figure 3:**
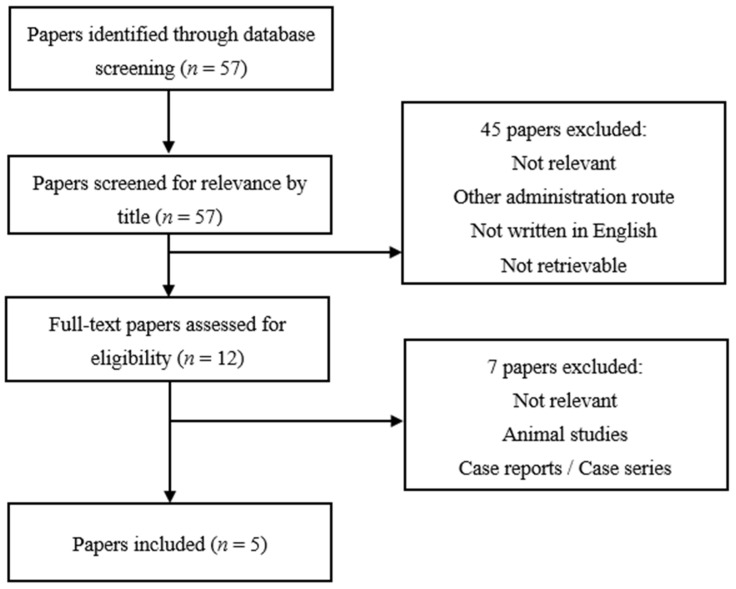
Study selection chart for systemic glucocorticoids.

We conducted a systematic literature search in Medline, using PubMed as the search engine. The search was performed for the last time on 14 February 2022. All papers identified through database screening were assessed for eligibility for inclusion independently by two review authors. The PRISMA 2020 flow diagram was used for the identification, screening and inclusion of articles, which is graphically depicted for each glucocorticoid administration route separately in Figure 1, Figure 2 and Figure 3. A detailed overview of MeSH-terms (medical subject headings) and search algorithms used is described in Table 1.

Before applying inclusion and exclusion criteria, this strategy yielded 38 results for intranasal glucocorticoids, 33 results for inhaled glucocorticoids and 57 results for systemic glucocorticoids. After the identification of these studies, they were screened for relevance, based on the PICO(TS) framework (patients, intervention, comparison, outcome, timing, setting) (Table 2). First, this was carried out by title and abstract, and for all articles considered relevant by title and abstract, a full-text assessment was carried out to determine eligibility for inclusion in this review. All original study types were included. Reviews, meta-analyses, case reports, case series and animal studies were excluded. Studies not published in English were also excluded.

Using the search terms mentioned above for the effect of intranasal glucocorticoids on IOP, the search yielded 38 results (Figure 1). Two other articles discussing the effect of intranasal glucocorticoids on IOP resulted from the search on inhaled glucocorticoids and were therefore also included here [16,17]. For intranasal glucocorticoids, we used two different combinations of search terms (Table 1) that yielded 8 overlapping studies, for which the duplicates were removed. The process of inclusion and exclusion of studies discussing intranasal glucocorticoids is demonstrated in Figure 1.

Using the search terms listed above for the effect of inhaled glucocorticoids on IOP, the search in Medline resulted in 33 studies (Figure 2). Two relevant papers were not retrievable online, and therefore a paper copy was retrieved from the library of the Faculty of Medicine, KU Leuven, Leuven, Belgium [17,18]. The further process of study selection is demonstrated in Figure 2.

Using the search terms for systemic glucocorticoids listed in Table 1, we retrieved 57 papers. The further process of study selection is demonstrated in Figure 3.

## 3. Results

After applying the inclusion and exclusion criteria for the study selection, we included 14 studies that discuss the effects of intranasal glucocorticoids on IOP, 12 discussing inhaled glucocorticoids, and five discussing systemically administered glucocorticoids.

### 3.1. Intranasal Glucocorticoids

An overview of the articles that discuss intranasally administered glucocorticoids is depicted in Table 3. Among the 14 included studies, 11 did not show any correlation between the use of intranasal glucocorticoids and an increased IOP [16,17,19,20,21,22,23,24,25,26,27]. In contrast to this finding, Bui et al. (2005) retrospectively reviewed twelve glaucoma patients taking intranasal glucocorticoids and found that the average IOP increased by 2.6 mmHg during steroid treatment compared with the pre-steroid examination (*p* = 0.007) [28]. In addition, after stopping the treatment with intranasal glucocorticoids, they observed a significant decrease in IOP (*p* = 0.011) [28]. The cross-sectional study conducted by Manji et al. in 2017 also suggests there is an increased risk of IOP elevation in long-term users of intranasal budesonide (administered daily for at least six months) [29]. Six percent of patients showed an increased IOP, although no significance level was mentioned [29]. More recently, the cross-sectional study by Mohd Zain et al. (2019) showed a significantly higher IOP in prolonged users of intranasal glucocorticoids for allergic rhinitis [30]. The rise of IOP was small (1.3 mmHg with a 95% confidence interval (CI) (0.72–1.9)), and no differences were shown in the cup–disc ratio. Exact treatment doses were not mentioned, but all patients received one or two puffs of intranasal momethasone, fluticasone or beclomethasone for an average of 5.42 years.

### 3.2. Inhaled Glucocorticoids

In Table 4, all included articles that discuss inhaled glucocorticoids are shown, among which three show an association with increased IOP. Mitchell et al. (1999) demonstrated an association between the use of inhaled glucocorticoids and an increased IOP in patients with a family history of glaucoma (odds ratio (OR) 3.1 with 95% CI (1.3–7.6)), although this association was not confirmed for people without such a family history [31]. Garbe et al. conducted a large case control study in 1997, showing a significantly increased risk of ocular hypertension or glaucoma in patients receiving high doses of inhaled glucocorticoids for at least three months continuously (OR 1.44 with 95% CI (1.01–2.06)). More recently, the cross-sectional case control study by Shroff et al. (2018) showed a higher IOP in chronic users of inhaled glucocorticoids (800 µg Budesonide or equivalents) compared to controls [32]. The difference in IOP was statistically significant (*p* < 0.001), although it was small: the observed pressure was 15.31 ± 3.27 mmHg for the inhaled glucocorticoid group versus 13.39 ± 1.95 mmHg for the control group. The study conducted by Nath et al. in 2017 showed 57 out of 405 subjects to have had an IOP higher than 22 mmHg after the intake of inhaled glucocorticoids, although no mention of statistical significance was made [33]. The eight remaining articles did not show any significant effect of inhaled glucocorticoids on IOP [11,18,34,35,36,37,38,39].

### 3.3. Systemic Glucocorticoids

An overview of the included articles that discuss systemically administered glucocorticoids is depicted in Table 5. Four studies described a correlation between systemic glucocorticoids and an increased IOP. Prasad et al. (2019) prospectively observed 33 children with auto-immune hepatitis, for whom a treatment with systemic prednisone was started at the time of diagnosis [40]. An elevated IOP, defined as a value of ≥20 mmHg or an elevation of ≥6 mmHg compared to baseline IOP, was observed in 20 children (61%) after one month of treatment (*p* < 0.001). There was no difference in initial prednisone dose or total cumulative dose for patients who did or did not present with an elevated IOP [40]. Second, Kaur et al. (2016) retrospectively reviewed 150 patients of a pediatric glaucoma clinic and found that 36 (24%) cases were steroid-induced [41]. However, they included patients receiving topical or oral glucocorticoids, and only 12 received oral glucocorticoids alone. No significantly different effect on IOP was shown between orally and topically administered glucocorticoids [41]. Garbe et al. (1997) performed a retrospective case control study that proved IOP to be elevated compared to baseline in current users of oral glucocorticoids older than 65 (OR 1,41 with 95% CI (1.22–1.63)) [42]. They also discovered a dose–response relationship, in which the increase in IOP was narrowly significant for daily doses under 80 mg of hydrocortisone (OR 1.26 with 95% CI (1.01–1.56) for doses under 40 mg and OR 1.37 with 95% CI (1.06–1.76) for doses from 40 to 80 mg), but the response became clearer at daily doses higher than 80 mg (OR 1.88 with 95% CI (1.40–2.53)) [42]. Finally, in the cross-sectional study performed by Gaur et al. in 2014, 11% of the examined children with nephrotic syndrome developed an increased IOP after receiving oral glucocorticoids for at least six months [43]. There was no significant association between the administered dose or the duration of glucocorticoid intake and raised IOP [43]. Only cumulative glucocorticoid doses are mentioned in this study, which means that the exact dose delivered on a daily basis remains unclear.

Only one study did not show a correlation between the intake of systemic glucocorticoids and an increased IOP. Gomes et al. (2014) found no correlation in patients with mixed connective tissue disease (MCTD) treated with low doses of prednisone (<10 mg daily for at least 6 months) [44].

## 4. Discussion

Since 1951, glucocorticoids are known to have the side effect of causing an increased IOP [3]. In contrast to topical ocular glucocorticoids, which are well known to cause a steroid response in a significant part of the general population [9,10], it is much less clear whether the same effect is to be expected for patients using intranasal, inhaled, or systemic glucocorticoids. A number of disquieting case reports on this topic have been published in the past, raising concerns about the possible ocular side effects following the administration of steroids by these routes. Opatowsky et al. (1995) described three patients, aged 60, 61 and 71, that developed ocular hypertension after starting therapy with beclomethasone diproprionate, administered by inhalation or nasal spray [45]. Second, Desnoeck et al. (2001) reported the case of an eight-year-old girl with bronchial asthma, treated with budesonide nasal spray 100 µg/day and budesonide inhalator 200 mg/day, in which ocular hypertension was discovered after two years of therapy [46]. Tham et al. (2004) described the case of a nine year old girl with leukemia that developed ocular hypertension after taking oral dexamethasone for only eight days [47]. Almost all patients described returned to an IOP within normal range after discontinuation of the glucocorticoid alone; only one patient needed IOP-lowering eyedrops. In addition to these examples, multiple other case reports and case series on the subject have been published [48,49,50,51]. These reports suggest the need for clear clinical guidance regarding the ophthalmological follow-up of glucocorticoid users. This review provides a relevant overview of the existing literature on the subject up until 2022 and serves as a first step toward a guideline for clinical practice.

### 4.1. Intranasal Glucocorticoids

Intranasal glucocorticoid administration specifically targets the nasal mucosa, which is the site where maximal drug effects are intended. As for all other topical administration forms, high local concentrations can be obtained without administering high systemic doses, and the amount of systemic adverse effects correlates with the drug fraction eventually reaching the systemic circulation. For intranasal glucocorticoids, this depends mostly on the absorption from the gastro-intestinal tract mucosa after swallowing [7]. The extent to which absorption from the upper airway mucosa contributes to the fraction reaching systemic circulation is almost negligible: Daley-Yates et al. (2001) measured a bioavailability of 44% for beclomethasone monopropionate, which fell to less than 1% after the administration of oral charcoal to exclude gastro-intestinal absorption [52]. This low absorption fraction from the upper airway mucosa can be explained by both the mucociliary transport toward the nasopharynx and the relatively small absorption surface [7]. The bioavailability of intranasally administered glucocorticoids depends on both the intestinal absorption and the liver’s first pass effect, and it varies from under 1% (for fluticasone propionate) to 41% (for beclomethasone propionate) [7].

Different administration modalities are available for the use if intranasal glucocorticoids, among which intranasal sprays, intranasal drops, and high-volume intranasal irrigation solutions are most widely used. Although the efficacy of these different administration forms can be similar for certain diseases, one should always consider every patient individually to determine the most appropriate regimen, based on factors such as the inflammation phenotype, bioavailability, dosage, cost, tolerability and side effects [53].

Among the fourteen articles included in our review that discuss the use of intranasal glucocorticoids, twelve describe an administration by nasal sprays, of which nine show no correlation with increased IOP. Manji et al. (2017) noticed a possible correlation in their cross-sectional study, however they did not mention statistical significance [29]. Only two studies report a significant effect of intranasal glucocorticoids on IOP. Bui et al. (2005) was the first study to report this, although some study characteristics need to be taken into account. Their study sample consisted of only twelve patients, making it the second smallest sample of all fourteen included studies. Patients were also taking a wide variety of nasal glucocorticoid sprays with different potencies and in different doses, making it impossible to draw straightforward conclusions from this study alone. The more recent cross-sectional study of Mohd Zain et al. (2019) reports a significantly higher IOP in patients with allergic rhinitis, treated chronically with intranasal glucocorticoids (mean 5.42 years, standard deviation 3.22 years). The observed difference in IOP was—however significant—very small (1.30 mmHg, 95% CI (0.72–1.90)). Moreover, no significant differences in vertical cup–disc ratio were noticed; thus, the clinical relevance of this small IOP elevation can be debated.

The remaining two studies concerning intranasal glucocorticoids describe patients receiving high-volume intranasal irrigations, in which glucocorticoids were added to a 240 mL saline solution [22,24]. None of the studies discussing these irrigations showed an association with raised IOP.

As twelve out of the fourteen included studies do not show any significant association between the administration of intranasal glucocorticoids and elevated IOP, and considering the pharmacokinetic properties of intranasal glucocorticoids, we conclude that they can be used safely in clinical practice. Generally, no supplementary ophthalmological controls are needed, although clinicians should always consider each patient individually at the commencement of therapy, and risk factors for steroid response (such as pre-existing glaucoma) should be taken into consideration.

### 4.2. Inhaled Glucocorticoids

Inhaled glucocorticoids are administered topically to the lower airway mucosa, and the fraction reaching systemic circulation depends both on the absorption from the gastro-intestinal tract mucosa and from the lower airway mucosa [7]. When using inhaling devices, a substantial part of the medication dose is not inhaled but deposited into the oropharynx and swallowed afterward, to be absorbed by the gastro-intestinal tract mucosa. The extent to which both mechanisms play a role depends on the extent of pulmonary deposition, and on whether or not a correct inhalation technique is used [7]. This implies large interindividual differences of glucocorticoids reaching systemic circulation after inhaled administration.

Eight out of the twelve included studies discussing inhaled glucocorticoids do not show an association with an increased IOP. In contrast, Nath et al. (2017) noticed the possibility for an increased IOP in COPD (chronic obstructive pulmonary disease) patients receiving inhaled glucocorticoids, although their results were not marked as statistically significant [33]. Among all included patients, 16.0% developed an IOP higher than 22 mmHg, and 3.92% developed damage to the optic nerve head [33]. They described a dose–response relationship, with the highest prevalence of glaucoma among the patients in the high-dose group (501–1000 µg of fluticasone propionate equivalents daily) [33]. Mitchell et al. (1999) reported an elevated IOP in users of inhaled glucocorticoids with a family history of glaucoma, an association that was not confirmed in individuals without such family history [31]. Furthermore, Garbe et al. (1997) showed a significantly increased risk for IOP elevation in patients who had been continuously taking high doses of inhaled glucocorticoids for at least three months [17]. In contrast, no increased risk was observed for patients receiving low to medium doses of inhaled glucocorticoids [17]. Despite these results, previous oral glucocorticoid intake was not taken into account. Second, the glucocorticoid doses that posed an increased risk of ocular hypertension were much higher than those generally prescribed in daily practice, wherefore the results may not be clinically relevant for the majority of individual patients [54]. Finally, the study by Shroff et al., in 2019, shows a small but significant increase in chronic users of lower doses of intranasal glucocorticoids [32]. The question arises whether this small increase in IOP is clinically relevant and will trigger glaucomatous progression, but the results of this study certainly justify additional ophthalmological control visits in certain patients with glaucoma or glaucoma suspects, when they are long-term users of (moderately) high doses of inhaled glucocorticoids.

Combining all these results and considering the pharmacokinetic properties of inhaled glucocorticoids, we can conclude that they can be used safely for most patients in most circumstances. Extra precautions should be taken when prescribing high doses of inhaled glucocorticoids or for patients with a family history of glaucoma. The extent to which a family history of glaucoma contributes to a patient’s predisposition to develop a steroid response following glucocorticoid inhalation still requires further investigation. Ophthalmological follow-up for IOP monitoring is recommended for these patients.

### 4.3. Systemic Glucocorticoids

Systemically administered glucocorticoids are expected to cause an increased IOP more often than intranasal or inhaled glucocorticoids because of higher doses reaching systemic circulation. In this case, not only the degree of side effects, but also the beneficial therapeutic effects depend on the systemic concentration that is reached [7].

Surprisingly, only a few studies on the subject have been published, varying greatly regarding patient age and glucocorticoid dosage. Five articles were retrieved, of which only the study by Gomes et al. (2014) did not demonstrate a correlation between the intake of systemic glucocorticoids and raised IOP [44]. The glucocorticoid doses administered in this study were low: all included patients were treated with less than 10 mg of prednisone equivalents daily. Among the included studies showing an association between systemic glucocorticoid intake and raised IOP, Kaur et al. (2016) [41] and Gaur et al. (2014) [43] did not mention daily doses. Prasad et al. (2019) mentioned a high incidence of IOP elevation in children treated with prednisone for auto-immune hepatitis [40]. Finally, Garbe et al. conducted a large case control study in 1997, in which a clear dose–response relationship was reported: the increase in IOP for daily doses under 80 mg of hydrocortisone equivalents was narrowly significant, but response became clearer at higher doses [42].

Since there are only a few articles discussing the IOP-related side effects of systemic glucocorticoids, caution is required when interpreting these results. Clinicians should be aware that patients receiving systemic glucocorticoids are at risk of developing an increased IOP. The highest risk is reported in users of high doses of glucocorticoids (>80 mg of hydrocortisone equivalents daily), whereas for low loses (<40 mg daily), the literature is contradictory. For every patient starting treatment with systemic glucocorticoids, especially children, regular ophthalmologic follow-up is warranted to detect steroid responders. Long-term systemic glucocorticoid users should also regularly be monitored for IOP elevation.

### 4.4. Glucocorticoids and Pre-Existing Glaucoma

Given that patients with pre-existing primary open-angle glaucoma (POAG) have a higher chance of being steroid-responders for topical intraocular glucocorticoids [10,11], the question arises of whether they are also more susceptible to an increased IOP caused by intranasal, inhaled, or systemic glucocorticoids. Among the articles discussed in this review, only four studied patients with pre-existing glaucoma. Regarding the effect of intranasal steroids, Bui et al. (2005) found a significant IOP elevation in intranasal steroid users with pre-existing glaucoma [28], although this association was denied by Yuen et al., in 2013 [21]. Both studies had small patient sample sizes, where definite conclusions cannot be drawn. The only two studies to discuss the effect of inhaled glucocorticoids on IOP in glaucoma patients both state that the risk of being a steroid responder does not increase [11,35]. Although no included articles discuss the use of systemic glucocorticoids in patients with pre-existing glaucoma, the phenomenon of a steroid response is especially important to diagnose in this patient group. If left unrecognized, even a small IOP elevation above the individual target pressure can induce progressive visual field defects and irreversible optic nerve head damage in glaucoma patients. Since patients with pre-existing glaucoma have a higher (up to 90%) risk of being a steroid responder, it is important to follow these patients on a regular basis at the start of their therapy. To determine whether patients with pre-existing POAG are at a higher risk of developing an increased IOP caused by intranasal or inhaled glucocorticoid administration forms, more research is needed.

## 5. Conclusions

The current literature indicates that patients receiving systemic glucocorticoids are at risk of developing an increased IOP, especially patients taking high daily doses. Regular ophthalmologic controls are therefore recommended for chronic steroid users and for patients starting with a new steroid treatment, especially for those with pre-existing glaucoma. Inhaled glucocorticoids may be associated with an increased IOP when delivered in high doses or in patients with a family history of glaucoma. Intranasal glucocorticoids have no clear IOP-elevating effect and can therefore be used safely without ophthalmologic follow-up in most circumstances. Clinicians should always consider each patient individually at the commencement of corticosteroid therapy in any form, and potential risk factors for a steroid response should be evaluated.

## Figures and Tables

**Table 1:** Search algorithms for each category of glucocorticoid administration.

Administration Form	Search Algorithm
Intranasal glucocorticoids	
Search 1	(“Administration, Intranasal”(Mesh) OR “Nasal Sprays”(Mesh) OR “Nasal Lavage”(Mesh)) AND (“Glucocorticoids”(Mesh) OR “Steroids”(Mesh)) AND “Intraocular Pressure”(Mesh)
Search 2	(“Rhinitis/drug therapy”(Mesh) OR “Sinusitis/drug therapy”(Mesh)) AND (“Glucocorticoids”(Mesh) OR “Anti-Inflammatory Agents”(Mesh) OR “Steroids”(Mesh)) AND (“Intraocular pressure”(Mesh) OR “eye/drug effects”(Mesh) OR “glaucoma”(Mesh) OR “ocular hypertension”(Mesh))
Inhaled glucocorticoids	
Search 1	(“Administration, Inhalation”(Mesh) OR “Nebulizers and Vaporizers”(Mesh) OR “Respiratory Therapy”(Mesh) OR “Respiratory Tract Absorption”(Mesh)) AND (“Glucocorticoids”(Mesh) OR “Steroids”(Mesh)) AND (“Intraocular Pressure”(Mesh) OR “glaucoma”(Mesh) OR “ocular hypertension”(Mesh))
Systemic glucocorticoids	
Search 1	(“Administration, Oral”(Mesh) OR “Capsules”(Mesh) OR “Tablets”(Mesh)) AND (“Glucocorticoids”(Mesh) OR “Steroids”(Mesh)) AND (“Intraocular Pressure”(Mesh) OR “glaucoma”(Mesh) OR “ocular hypertension”(Mesh))

**Table 2:** PICO(TS) framework for the literature search.

Patients	People with any medical condition requiring intranasal, inhaled or systemic glucocorticoid therapy.
Intervention	A treatment with intranasal, inhaled or systemic glucocorticoids.
Comparison	No treatment with intranasal, inhaled or systemic glucocorticoids.
Outcome	Intraocular pressure elevation.

**Table 3:** Overview of the articles discussing intranasal glucocorticoid administration.

Study	Study Type (Evidence Level)	Patients Included	Age (Years)	Steroid + Daily Dose	IOP Increase?
Mohd Zain et al., 2019	Cross-sectional case control (3B)	95	10–40	Momethasone Fluticasone Beclomethasone	Yes
Bui et al., 2005 *	Retrospective Chart Review (4)	12	35–83	variable	Yes
Manji et al., 2017	Cross-sectional observational (4)	100	>19	Budesonide 500 µg	Possible
Martino et al., 2015	Retrospective descriptive (4)	10	15–85	Dexamethasone 800 µg	No
Yuen et al., 2013 *	Randomized Controlled Trial (1B)	19	18–85	Beclomethasone 400 µg	No
Man et al., 2013	Prospective observational (4)	23	>18	Fluticasone 3000 µg ^a^	No
LaForce et al., 2013	Randomized Controlled Trial (1B)	548	>12	Fluticasone 110 µg	No
Seiberling et al., 2013	Prospective observational (4)	18	>18	Budesonide 500 µg ^a^	No
Ozkaya et al., 2011	Cross-sectional case control (3B)	240	7–15	Budesonide 100 µg	No
Spiliotopoulos et al., 2007	Prospective observational (4)	54	22–55	Dexamethasone 20 µg	No
Chervinsky et al., 2007	Randomized Controlled Trial (1B)	663	≥12	Ciclesonide 200 µg	No
Bross-Soriano et al., 2004	Prospective comparative (4)	360	18–60	Fluticasone 200 µg Mometasone 200 µg Beclomethasone 400 µg	No
Öztürk et al., 1998	Prospective observational (4)	26	18–66	Budesonide 400 µg Beclomethasone 400 µg	No
Garbe et al., 1997	Retrospective case control (3B)	48,118	>66	Fluticasone < or ≥200 µg Flunisolide < or ≥200 µg Beclomethasone < or ≥400 µg Budesonide < or ≥400 µg Triamcinolone < or ≥400 µg	No

* Studies including patients with pre-existing glaucoma. ^a^ Glucocorticoid doses were added to a 240 mL saline solution for administration by intranasal irrigation.

**Table 4:** Overview of the articles discussing inhaled glucocorticoid administration.

Study	Study Type (Evidence Level)	Patients Included	Age (Years)	Steroid + Daily Dose	IOP Increase?
Shroff et al., 2018	Cross-sectional case control (3B)	400	18–89	Budesonide 800 µg or equivalents	Yes
Mitchell et al., 1999	Cross-sectional observational (4)	3654	49–97	Beclomethasone ≤2 puffs >2 to ≤4 puffs >4 puffs	Yes ^a^
Garbe et al., 1997	Retrospective case control (3B)	48,118	>66	Low versus high dose exposure: Beclomethasone < or ≥1600 µg Budesonide < or ≥1600 µg Triamcinolone < or ≥600 µg Flunisolide < or ≥1500 µg	Yes ^b^
Nath et al., 2017	Prospective observational (4)	405	>50	Fluticasone equivalents ^c^	Possible
Kerwin et al., 2019	Randomized Controlled Trial extension (1B)	456	40–80	Budesonide 320 µg	No
Moss et al., 2017 *	Randomized Controlled Trial (1B)	22	18–85	Fluticasone 500 µg	No
Alsaadi et al., 2012	Prospective observational (4)	93	5–15	Fluticasone 250 µg	No
Johnson et al., 2012 *	Retrospective case control (3B)	170	Not specified	Not specified	No
Gonzalez et al., 2010	Retrospective case control (3B)	15,736	≥66	Fluticasone equivalents ^d^	No
Behbehani et al., 2005	Prospective observational (4)	95	<12	Budesonide 100–1050 µg Beclomethasone 100–1050 µg	No
Duh et al., 2000	Randomized Controlled Trials (1B)	1255	6–70	Budesonide 200–1600 µg	No
Samiy et al., 1996	Prospective observational (4)	187	20–79	Not specified	No

^a^ IOP elevation only in patients with a family history of glaucoma. ^b^ IOP elevation only in patients receiving high doses continuously for at least 3 months. ^c^ Doses of different glucocorticoids were expressed as fluticasone equivalents: Low: 1–250 µg; Intermediate: 251–500 µg; High: 501–1000 µg. ^d^ Doses of different glucocorticoids expressed as fluticasone equivalents: Low: <500 µg; Intermediate: 500–999 µg; High: ≥1000 µg. * Studies including patients with pre-existing glaucoma.

**Table 5:** Overview of the articles discussing systemic glucocorticoid administration.

Study	Study Type (Evidence Level)	Patients Included	Age (Years)	Steroid + Daily Dose	IOP Increase?
Prasad et al., 2019	Prospective cohort (2B)	33	1–18	Prednisone 1–2 mg/kg/day, tapered after 2–4 weeks	Yes
Kaur et al., 2016	Retrospective observational (4)	150	<12	Not specified	Yes
Gaur et al., 2014	Cross- sectional observational (4)	82	4–18	Not specified	Yes
Garbe et al., 1997	Cross-sectional case control (3B)	48,118	>65	Hydrocortisone equivalents ^a^	Yes
Gomes et al., 2014	Cross- sectional case control (3B)	106	>18	Variable, expressed as prednisone equivalents <10 mg	No

^a^ Doses of different glucocorticoids were expressed as hydrocortisone equivalents: Low: <40 mg; Intermediate: 40–79 mg; High: ≥80 mg.

## Data Availability

Not applicable.
